# The efficacy of recombinant feline interferon-omega in treating symptomatic cats infected with feline immunodeficiency virus

**DOI:** 10.18849/ve.v8i3.666

**Published:** 2023-09-15

**Authors:** Rachel Garrett+

**Keywords:** CATS, FELINE IMMUNODEFICIENCY VIRUS, FIV, RETROVIRUS, INTERFERON-OMEGA, rFeIFN-ω, VIRBAGEN® OMEGA, THERAPY

## Abstract

**PICO question:**

In symptomatic cats with feline immunodeficiency virus (FIV), does high-dose subcutaneous recombinant feline interferon-omega (rFeIFN-ω) administration lead to reduced clinical signs compared to cats who were not administered rFeIFN-ω?

**Clinical bottom line:**

**Category of research:**

Treatment.

**Number and type of study designs reviewed:**

Three studies were critically reviewed, including one randomised controlled trial, one non-randomised controlled trial, and one uncontrolled clinical trial.

**Strength of evidence:**

Weak.

**Outcomes reported:**

Each of the studies reported that rFeIFN-ω administration significantly reduced clinical signs in FIV infected cats. However, all three papers have limitations in their study design and statistical analysis which lower the strength of the evidence they provide.

**Conclusion:**

There is only weak evidence demonstrating that rFeIFN-ω administration leads to reduced clinical signs in FIV positive cats. Currently, there is a lack of well-designed, double-blinded, randomised, placebo-controlled clinical trials which have an adequate sample size and specifically focus on FIV positive cats. As a result, more robust evidence is needed to prove the definitive therapeutic benefit of rFeIFN-ω in symptomatic FIV patients. Treatment with rFeIFN-ω may still be considered by clinicians for cats with clinical signs potentially associated with retroviral infection, such as oral lesions and stomatitis, particularly if they are not responding well to supportive therapy alone.

## How to apply this evidence in practice

The application of evidence into practice should take into account multiple factors, not limited to: individual clinical expertise, patient’s circumstances and owners’ values, country, location or clinic where you work, the individual case in front of you, the availability of therapies and resources.

Knowledge Summaries are a resource to help reinforce or inform decision making. They do not override the responsibility or judgement of the practitioner to do what is best for the animal in their care.

## Clinical scenario

An 11-year-old, male domestic short-haired cat is presented to you with gingivostomatitis and weight loss. Due to his history of getting into frequent cat fights, you run a point-of-care feline immunodeficiency virus (FIV) / feline leukaemia virus (FeLV) test, and it returns an antibody positive result for FIV. You advise the owner that their cat would benefit from lifestyle changes and supportive therapy, such as dental assessment and cleaning. However, the owner asks if there is any medication available to directly target the cat’s FIV infection and better resolve his clinical signs. You have heard of the use of recombinant feline interferon-omega (rFeIFN-ω) for treating cats with retroviral infections, however you are unsure of how efficacious it is for improving clinical signs in symptomatic FIV patients.

## The evidence

Three studies relevant to this Knowledge Summary’s PICO question were identified, including a randomised, double-blinded, placebo-controlled trial (de Mari et al., 2004), a non-randomised controlled trial (Doménech et al., 2011), and an uncontrolled clinical trial (Gil et al., 2013). All three papers had consistent findings regarding the efficacy of rFeIFN-ω for the treatment of symptomatic FIV patients, however the strength of the evidence provided by each study differs.

The de Mari et al. (2004) trial has the best study design with the strongest level of evidence, as it is the only randomised controlled trial out of the three papers. However, it is limited by the fact that all of the FIV positive cats in the trial were coinfected with FeLV, which potentially confounds the study results in relation to this PICO question. Furthermore, the Doménech et al. (2011) trial also employed a control group, however it suffers from the fact that it was not randomised, blinded, or placebo-controlled. Out of the three papers, the final trial by Gil et al. (2013) has the weakest study design due to its lack of a control group, making the study results more prone to bias. Regardless of the study design, all of the papers had a small sample size of FIV positive cats, hence negatively impacting the power of the results in regard to this PICO question.

## Summary of the evidence

**de Mari et al. (2004):** 

Population:	Feline leukaemia virus (FeLV) / feline immunodeficiency virus (FIV) naturally coinfected cats and FeLV naturally infected cats. Inclusion criteria: Symptomatic cats, with at least one clinical sign potentially related to infection (e.g. pyrexia, anorexia, altered behaviour, polydipsia, dehydration, pale mucous membranes, stomatitis). Enzyme-linked immunosorbent assay (ELISA)-positive blood tests for FeLV / FIV (SNAP Combo FeLV / FIV kit, IDEXX).
Sample size:	81 cats: FeLV positive: n = 57; FeLV / FIV copositive: n = 24.
Intervention details:	The cats were randomly assigned into two groups: a recombinant feline interferon-omega (rFeIFN-ω) treatment group and a placebo group. The owners and veterinarians were both blinded to the treatment each cat was receiving. The treatment group (n = 39) was given three courses of subcutaneous rFeIFN-ω (Virbagen^® ^Omega, Virbac) injections from days 0–4, 14–18, and 60–64. Each treatment course consisted of 1 MU/kg subcutaneous injection once daily for 5 consecutive days. The placebo group (n = 42) followed the same schedule, however they were injected with a placebo instead. During the study, all cats also received individual supportive therapies if indicated (e.g., fluid therapy, vitamins, antibiotics if there was suspected bacterial infection, non-steroidal anti-inflammatory drugs (NSAIDs), etc.). Corticosteroids were not permitted due to their immunomodulatory effects.
Study design:	Randomised, double-blinded, placebo-controlled trial.
Outcome studied:	Seven categories of clinical signs often associated with retroviral infection were monitored on days 0, 14, 30, 60, and 120: rectal temperature, general behaviour, appetite, thirst, dehydration, mucous membrane appearance, and stomatitis. A severity rating ranging from 0–3 was assigned to each category. At each time point, the ratings in every category were summated to form overall clinical scores (CS) for each animal. The CS were then compared between time points to track improvement in clinical signs over time.
Main Findings (relevant to PICO question)	On day 0, the CS of the treatment and placebo groups were not significantly different. Immediately after treatment, cats in both groups experienced rapid and general improvement of clinical signs. However, the cats in the rFeIFN-ω treatment group had consistently lower CS than the placebo group across all time points (P = 0.049), indicating a significant reduction in clinical signs compared to the placebo group.
Limitations:	Separate statistical analysis was not performed to assess improvement in CS in the FeLV versus the FIV / FeLV groups of cats. Therefore, it is unknown whether the clinical improvement in the FIV / FeLV cats following treatment was different from the clinical improvement in the FeLV cats.

**Doménech et al. (2011):** 

Population:	Feline immunodeficiency virus (FIV) and feline leukaemia virus (FeLV) naturally infected cats. Inclusion criteria: Both asymptomatic and symptomatic cats were included.
Sample size:	21 cats: FIV positive: n = 12. FeLV positive: n = 9.
Intervention details:	Cats were non-randomly allocated into a treatment and a control group: Treatment group (n = 11) (household cats): four FeLV positive cats, seven FIV positive cats. Control group (n = 10) (cats housed in a cattery): five FeLV positive cats, five FIV positive cats. The treatment group was given commercial recombinant feline interferon-omega (rFeIFN-ω [Virbagen^®^ Omega, Virbac]) according to the licensed protocol, consisting of three courses of subcutaneous injections from days 0–4, 14–18, and 60–64. Each treatment course consisted of 1 MU/kg subcutaneous injection once daily for 5 consecutive days. The control group was left untreated, and not provided with a placebo.
Study design:	Non-randomised controlled trial.
Outcome studied:	Prior to the treatment course, each cat was examined for clinical signs commonly associated with retroviral infection: loss of appetite, weakness, dehydration, weight loss, lymphadenomegaly, pale mucous membranes, polyuria / polydipsia, conjunctivitis, keratitis, oral lesions, digestive disorders, cutaneous lesions, respiratory disorders, neurologic disorders, lymphoma, myeloproliferative disorders, and other neoplasia. Each clinical sign was given a severity rating from 0–2, and these ratings were added together to form overall clinical scores (CS). Cats with a CS ≥ 6 (10/21 [48%]) were considered to have severe disease. 3/21 (14%) cats were asymptomatic, with a CS of 0. The CS in each cat was reassessed 2 weeks after the treatment course, and compared to the original CS in order to assess clinical improvement.
Main Findings (relevant to PICO question)	After the rFeIFN-ω treatment course, a statistically significant clinical improvement was observed in all symptomatic cats in the treatment group in comparison to the control group (P <0.05). The most significant improvement was seen in cats who started the study with CS of ≥6. Asymptomatic cats or those with mild disease remained stable.
Limitations:	The method of FeLV and FIV diagnosis was not specified. The study was not blinded. The study was not randomised, and no statistical analysis was conducted at the beginning of the study to ensure the initial CS were similar in the treatment versus the control cats. No placebo was provided to the control group. The control group was housed in a cattery, and therefore they were exposed to a more stressful environment than the household cats in the treatment group. This could confound the results and introduce bias. The study had a small sample size, particularly when only considering the FIV positive cats. Separate statistical analysis was not performed to assess improvement in CS in the FeLV versus the FIV groups of cats. Therefore, it is unknown whether the clinical improvement in the FIV cats following treatment was different from the clinical improvement in the FeLV cats.

**Gil et al. (2013):** 

Population:	Feline immunodeficiency virus (FIV), feline leukaemia virus (FeLV), and FIV / FeLV naturally infected cats Inclusion criteria: At least one clinical sign potentially related to retroviral infection (e.g., pyrexia, anorexia, altered behaviour, polydipsia, dehydration, pale mucous membranes, stomatitis). Positive ELISA blood test for FIV, FeLV, or both (ViraCHEK^®^ FIV and ViraCHEK^®^ FeLV, Synbiotics).
Sample size:	16 cats: FIV positive: n = 7. FeLV positive: n = 6. FIV / FeLV positive: n = 3.
Intervention details:	All cats (n = 16) were treated with commercial rFeIFN-ω (Virbagen^®^ Omega, Virbac) according to the licensed protocol, consisting of three courses of subcutaneous injections from days 0–4, 14–18, and 60–64. Each treatment course consisted of 1 MU/kg subcutaneous injection once daily for 5 consecutive days. Cats were also provided with supportive treatment during therapy if indicated; potentiated amoxicillin, hepatic protectants, and fluid therapy. However, antibiotics other than potentiated amoxicillin, corticosteroids, and non-steroidal anti-inflammatory drugs (NSAIDs) were not permitted in order to avoid potential immunomodulation.
Study design:	Uncontrolled clinical trial.
Outcome studied:	At days 0, 10, 30 and 65, all cats were assessed for 11 clinical signs commonly associated with retroviral infection: oral ulcers / gingivitis, caudal stomatitis / palatitis, ophthalmic abnormalities, lymphadenopathy, ocular and nasal discharge, mucous membrane colour, coat appearance, body condition score, faecal appearance, and concurrent diseases / co-morbidities. Each of these clinical signs was given a severity rating from 0–2, and all of these ratings were summated to form overall clinical scores (CS) for each animal. The CS were then compared between each time point.
Main Findings (relevant to PICO question)	Oral ulcers / gingivitis and caudal stomatitis were the most frequent clinical signs observed at day 0 in all groups. These clinical signs also improved the most consistently throughout the treatment period, whereas changes in the other criteria were more variable. When considering all 16 cats, there was a significant improvement in the overall CS between days 0 and 60 (P <0.05). The improvement in CS was most pronounced in cats who started the study with higher initial CS. Ten cats had a reduction in clinical signs, whereas six cats maintained the same clinical status. No cats experienced a worsening of their condition. In the FIV positive group specifically (7/16 [44%]), there was also a statistically significant clinical improvement after treatment (P <0.05). Four of the seven FIV positive cats demonstrated marked improvement (final CS > 50% better than initial), one cat showed mild to moderate improvement (final CS up to 50% better than initial), and in two cats the CS remained unchanged.
Limitations:	The study had a small sample size, especially when only considering the FIV positive cats. There was no control group. There is a lack of variety in clinical signs in the study population, as the majority of the FIV positive cats had oral ulcers and caudal stomatitis as their primary clinical sign. One author was an employee of Virbac, who produce Virbagen^®^ Omega (commercial rFeIFN-ω), creating a potential conflict of interest.

## Appraisal, application and reflection

Current treatment of feline immunodeficiency virus (FIV) patients predominantly revolves around supportive therapy, such as controlling secondary infections with antimicrobial drugs (Dunham & Graham, 2008). However, symptomatic FIV patients may benefit from more targeted therapy. One such therapy is recombinant feline interferon-omega (rFeIFN-ω), which has the potential to reduce clinical signs due to its antiviral and immunomodulatory properties (Doménech et al., 2011; and Gerlach et al., 2009). Despite the fact that rFeIFN-ω has been licensed for use in multiple countries for several years, there are limited published in vivo studies which assess how efficacious it is at improving clinical signs in FIV infected cats. This Knowledge Summary aims to critically appraise the existing literature, in order to assist veterinarians in practicing evidence-based medicine when treating symptomatic FIV patients.

A literature search yielded three studies relevant to this PICO question (de Mari et al., 2004; Doménech et al., 2011; and Gil et al., 2013). Each of these studies reported that rFeIFN-ω administration significantly reduced clinical signs in FIV infected cats. However, all three papers had limitations in their study design and statistical analysis which should be considered when interpreting these findings.

None of the papers focused solely on the clinical benefits of rFeIFN-ω in symptomatic FIV positive cats, as all of them also included feline leukaemia virus (FeLV) positive cats in the study population. In both the de Mari et al. (2004) and Doménech et al. (2011) papers, the FeLV and FIV positive cats were not treated as distinct groups when the statistical analysis was conducted. Cats infected with FeLV typically develop more severe clinical signs than those with FIV (Leal & Gil, 2016); therefore, it is possible that including the FeLV cats in the same analysis as the FIV cats skewed the data in these studies, as it could have potentially elevated the average improvement in clinical scores in the treatment group. This issue is compounded by the fact that all of the FIV infected cats in the de Mari et al. (2004) study were also coinfected with FeLV. Based on these factors, it is unknown whether the reported improvement in clinical signs would still be statistically significant in the de Mari et al. (2004) and Doménech et al. (2011) papers if the FIV positive cats were considered separately from FeLV positive cats. Therefore, the specific clinical benefits of rFeIFN-ω treatment in FIV positive cats cannot be ascertained from these studies.

Gil et al. (2013) is the only study that did distinct statistical analysis for the FIV and FeLV positive cats. In this study, it was demonstrated that the FIV positive group specifically had a statistically significant improvement in clinical signs, with 4/7 (57%) cats demonstrating marked improvement. Oral ulcers / gingivitis and caudal stomatitis were the clinical signs that most consistently improved. This is promising data, however rFeIFN-ω can be effective in treating caudal stomatitis even in FIV negative cats (Matsumoto et al., 2018), and therefore the benefits reported by the study may not be applicable to other FIV disease presentations. Additionally, it must be noted that unlike the other two papers, the Gil et al. (2013) study did not include a control group. The study addressed this in its discussion and stated that the clinical scores of the cats at day 0 of treatment could act as the control for each cat. However, the perceived clinical improvement in the cats between day 0 and day 65 could be due to a variety of external confounding factors, such as the level of supportive care received by each cat throughout the study. Thus, without a control it is impossible to prove direct causation between the rFeIFN-ω treatment and the clinical improvement observed.

Conversely, both de Mari et al. (2004) and Doménech et al. (2011) included a control group in their studies. However, the study design of the Doménech et al. (2011) paper had its own shortcomings, as the treatment and control groups were not treated equally throughout the study. The trial was not randomised, and no statistical analysis was conducted at the beginning of the study to ensure the initial clinical scores were similar in the treatment versus the control cats. This introduces selection bias into the study, since it is unknown whether the two groups were equivalent at the beginning of the trial. Given there was a very wide range of initial clinical scores in the study cats, ranging from 0 (asymptomatic) to 6 and above (severe disease), this selection bias could have significantly influenced the perceived clinical improvement in the treatment group compared to the control. Moreover, the study personnel were not blinded. This leads to information bias, particularly since the clinical scoring method utilised in the study was largely subjective and dependent on the opinion of the assessor. The control group cats were also not provided with a placebo, and unlike the treatment group they were housed in a cattery. Since they experienced a different environment from the treatment group throughout the duration of the study, it is possible external factors could have confounded the results, as management and housing conditions can impact FIV disease progression (Beczkowski et al., 2015). Thus, the Doménech et al. (2011) study did not take steps to adequately minimise bias, resulting in a reduction in its internal validity.

Out of the three studies, the de Mari et al. (2004) paper is the only one which is randomised, placebo-control and double-blinded, and thus it represents a stronger level of evidence than both the Doménech et al. (2011) and Gil et al. (2013) studies. However, as mentioned previously, the study population did not contain any cats that were solely infected with FIV, as all the cats were either FeLV infected or FeLV/FIV coinfected. Thus, the population of this study is the least relevant to this Knowledge Summary, and the clinical benefits of rFeIFN-ω demonstrated in the results cannot be extrapolated to FIV patients in practice unless they are also coinfected with FeLV.

Regardless of the study design, no papers provided sample size calculations, and all three studies suffered from a small sample size of FIV positive cats. The de Mari et al. (2004), Doménech et al. (2011) and Gil et al. (2013) studies only included 24, 12, and 10 FIV infected or FIV / FeLV coinfected cats, respectively. This negatively impacts the power of the study results in regards to this PICO question, hence leading to higher rates of random error and poor precision. Hence, veterinarians contemplating the use of rFeIFN-ω should consider that clinical improvement has yet to be reported in a study with a sufficiently large number of FIV positive cats enrolled.

Furthermore, all of the papers failed to consider whether the clinical signs experienced by each cat at the beginning of the study were truly due to FIV. The causative relationship between FIV and clinical disease is not well-established in the majority of circumstances, particularly for non-specific signs such as weight loss (White et al., 2011). Even for more specific clinical signs typically associated with FIV, for example oral lesions and stomatitis, it is still unclear whether FIV plays a major role in precipitating disease (White et al., 2011). Therefore, it is difficult to determine whether the clinical signs evaluated in each study were directly related to FIV infection, or whether they were due to age or other disease processes. As a result, the treatment efficacy of rFeIFN-ω for FIV is hard to accurately assess in these studies, and the inherent complexity of FIV disease associations should be considered when interpreting the results.

The cost of treatment and difficulty of administering subcutaneous injections in some cats may also be limiting factors for clinicians considering the use of rFeIFN-ω. An alternative option is oral administration, an off-label protocol which may be advantageous due to its ease of administration and cheaper cost when compared to the licensed subcutaneous protocol. A 2014 study by Gil et al. evaluated clinical improvement in symptomatic FIV positive cats who received the oral rFeIFN-ω protocol and used the results from the Gil et al. 2013 study on subcutaneous administration as the control group. No significant difference in clinical improvement was observed between the oral and subcutaneous routes of administration, and thus the oral off-label protocol may be a favourable treatment option in some circumstances (Gil et al., 2014). However, the 2014 Gil et al. study suffers from similar issues as the 2013 Gil et al. study evaluated in this Knowledge Summary, namely the lack of a placebo control, as well as a small sample size of only 11 FIV positive cats. Therefore, further critical appraisal of the evidence surrounding the oral protocol is required by clinicians who are considering this route of administration.

In conclusion, due to the limitations outlined above, there is only weak evidence demonstrating that rFeIFN-ω administration leads to reduced clinical signs in FIV positive cats. Although each paper reported that rFeIFN-ω significantly reduced clinical signs, more robust evidence is still needed to prove its definitive therapeutic benefit in symptomatic cats with FIV. Currently, there is a lack of well-designed, double-blinded, randomised, placebo-controlled clinical trials which have an adequate sample size and specifically focus on FIV positive cats; these factors should be considered as essential criteria for those undertaking future studies on the topic. Treatment with rFeIFN-ω may still be considered by clinicians for cats with clinical signs potentially associated with retroviral infection, particularly if they are not responding well to supportive therapy alone.

## Methodology

**Search strategy:** 

Databases searched and dates covered:	CAB Abstracts via Web of Science (1973–present) Medline via Ovid (1946–present) Web of Science Core Collections via Web of Science (1900–present)
Search strategy:	CAB Abstracts: cat OR cats OR feline OR felines FIV OR retrovir* OR immunodeficiency virus Interferon* OR rFeIFN* OR IFN* OR antiviral OR immunomodulat* OR treat* Omega 1 AND 2 AND 3 AND 4 Medline: Cats/ (MeSH) OR cat OR cats OR feline OR felines Immunodeficiency Virus, Feline/ (MeSH) OR FIV OR retrovir* OR immunodeficiency virus Interferon* OR rFeIFN* OR IFN* OR Interferons/ (MeSH**) **OR Antiviral Agents/ (MeSH) OR antiviral OR immunomodulat* OR Therapeutics/ (MeSH) OR treat* Omega 1 AND 2 AND 3 AND 4 Web of Science: cat OR cats OR feline OR felines FIV OR retrovir* OR immunodeficiency virus Interferon* OR rFeIFN* OR IFN* OR antiviral OR immunomodulat* OR treat* Omega 1 AND 2 AND 3 AND 4
Dates searches performed:	08 May 2023

**Exclusion / inclusion criteria:** 

Exclusion:	Not relevant to PICO question. Non-English studies. Literature reviews. Conference papers. Dossiers.
Inclusion:	Relevant to PICO question. English studies. Peer-reviewed, primary research studies including more than one cat.

**Search outcome:** 

Database	Number of results	Excluded – Not relevant to PICO	Excluded – Not in English	Excluded – Non-English studies	Excluded – Literature reviews, dossiers, conference proceedings	Total relevant papers
CAB Abstracts	27	15	3	5	1	3
Medline	14	9	0	2	0	3
Web of Science	43	33	0	5	2	3
Total relevant papers when duplicates removed						3

## ORCiD

Rachel Garrett: https://orcid.org/0000-0002-0939-7329

## Conflict of interest

The author declares no conflicts of interest.

## Disclaimer

Knowledge Summaries are a peer-reviewed article type which aims to answer a clinical question based on the best available current evidence. It does not override the responsibility of the practitioner. Informed decisions should be made by considering such factors as individual clinical expertise and judgement along with patient’s circumstances and owners’ values. Knowledge Summaries are a resource to help inform and any opinions expressed within the Knowledge Summaries are the author’s own and do not necessarily reflect the view of the RCVS Knowledge. Authors are responsible for the accuracy of the content. While the Editor and Publisher believe that all content herein are in accord with current recommendations and practice at the time of publication, they accept no legal responsibility for any errors or omissions, and make no warranty, express or implied, with respect to material contained within. For further information please refer to our Terms of Use.
